# Allelopathy: Mechanisms and Applications in Regenerative Agriculture

**DOI:** 10.3390/plants13233301

**Published:** 2024-11-25

**Authors:** Margot Schulz, Vincenzo Tabaglio

**Affiliations:** 1 IMBIO Institute of Molecular Physiology and Biotechnology of Plants, University of Bonn, Karlrobert-Kreiten Str. 13, 53115 Bonn, Germany; 2 Department of Sustainable Crop Production DI.PRO.VE.S., Section Agronomy and Plant Biotechnologies, Università Cattolica del Sacro Cuore, Via Emilia Parmense 84, 29122 Piacenza, Italy; vincenzo.tabaglio@unicatt.it

Allelopathy is an important mechanism in plant communication and interference, involving the release of plant/microorganism self-produced, special featured organic molecules into the environment. These molecules (allelochemicals) inhibit or stimulate the growth of neighbored plants and microorganisms (targets), depending on their type and dosage. They are therefore regarded as natural plant growth modulators, with the effects also influenced by the developmental and other characteristics of the target plants. When used as a form of negative interaction between species, allelopathy in agriculture serves as a proven, sustainable tool for integrated weed control in organic farming, thereby mitigating environmental risks, a key objective of the Green Deal and Farm to Fork Strategy of the EU Agricultural Policy. Allelopathy can reduce the use of synthetic herbicides or of chemically upgraded natural compounds which cannot be no longer considered natural products.

The adoption of allelopathy (plant-plant and plant-microbe interactions) in regenerative agricultural practices still requires thorough multidisciplinary research (encompassing chemistry, genetics, metabolism, and agronomy) to address two complementary areas of focus. First, screening is needed to identify new allelopathic plants and their allelochemicals. Second, it is crucial to investigate the agronomic feasibility of effectively using these plants and their metabolites in conventional agroecosystems through field studies. For the latter, it is important to assess the potential for incorporating these allelopathic plants into crop rotations, often as cover crops, without adversely affecting subsequent crops.

This special issue on allelopathy aims to gather current experimental findings from around the world, covering both basic and applied aspects. It serves as a convenient and accessible resource for researchers working in this field and may contribute to promote future research on allelopathic interactions.

It compiles fourteen articles (nine original research and five original reviews) covering different aspects of allelopathy. The original research articles present recent investigations on allelochemical identification, on tritrophic interaction, on molecular effects in plant growth modulation and on undesired pathogen propagation via allelochemicals.

As can be observed, there is a lack of articles in the more strictly agronomic significance, specifically regarding the application of these results in open-field conditions. This highlights the need for greater research efforts in this important area, which is certainly more costly from an experimental perspective but is absolutely necessary for transitioning from basic knowledge to applied knowledge.

Following, some key points of the research articles are listed.

Vieites-Álvarez et al. [1] characterize the chemical profile of shoots, roots, and root exudates of four buckwheat accessions, emphasizing on polyphenol accumulation and exudation. The buckwheat Gema (accession number 01Z5000112) appeared to be most promising for sustainably control of the two herbicide-resistant target weeds *Lolium rigidum* Gaud. and *Portulaca oleracea* L. Espinosa-Colín et al. [2] present phytotoxic propiophenone, 4-ethylacetophenone, and 2,4-dimethylacetophenone obtained from *Cistus ladanifer*. Using *Lactuca sativa* and *Allium cepa* as target plants, dosage dependency of inhibitory and hormetic effects of the compounds were investigated.

The study of Amri et al. [3] addresses the chemical composition of essential oils from *Eucalyptus falcata*, *E. sideroxylon* and *E. citriodora*. They determined remarkable antioxidant, antimicrobial and phytotoxic properties of the essential oils. Germination and growth of *Sinapis arvensis*, *Phalaris canariensis*, and *Triticum durum* seedlings were inhibited. Marques et al. [4] contribute with a work on phytotoxic, water-soluble quillaic acid-based triterpene saponins isolated from the leaves of *Quillaja lancifolia*. Kato-Noguchi et al. [5] investigated the hitherto unknown allelopathic properties of *Osmanthus fragrans*, *Osmanthus heterophyllus* and of a hybrid of these two species, *Osmanthus × fortunei* Carrière. Main allelopathic substances of *O. × fortunei* and *O. fragrans* were identified as (+)-pinoresinol and 10-acetoxyligustroside, which are released into the environment during decomposition.

The contribution of Preusche et al. [6] demonstrated the concentration-dependent influence of the *Mentha x piperita* volatilome, *Mentha* essential oil fraction and limonene on the expression profile of distinct TCP transcription factors that modulate *Arabidopsis thaliana* and cabbage leaf growth under different culture conditions. Staszek et al. [7] investigated the phytotoxic effect of the digestive fluid of *Nepenthes x ventrata* on tomato seed germination and seedlings root growth. Oxidative stress elicited by the digestive fluid is supposed to inhibit tomatoes. A study dealing with the trophic interaction between *Peudosphinx tetrio* L. larvae and the toxic Apocynaceae *Allamanda cathartica* L. is presented by Matignon et al. [8]. The chemical profiles of the leaves of healthy and herbivorous *A. cathartica* and of the excretions of the caterpillars was analyzed. Larval excretion of the bioactive compounds from the host plant is one possibility to escape the harmful effects of these compounds.

Xin et al. [9] present investigations on the interactions of the allelochemicals and pathogens of potato including *A. solani*, *B. cinerea*, *F. solani*, *F. oxysporum*, *C. coccodes*, and *V. dahlia*. 7-Methoxycoumarin had inhibitory effects but, for instance, caffeic acid and chlorogenic acid promoted the pathogens. The results led to the conclusion that autotoxic allelopathy and promotion of pathogens caused by the accumulation of distinct allelochemicals can result in replant problems of potato.

The five original reviews summarize important features of *Cyperus esculentus*, *Solidago species*, *Abutilon theophrasti*, and microalgae allelopathy, whereas the contribution of Lewerenz et al. [10] attends to translocation of allelochemicals between plants, emphasizing on pyrrolizidine alkaloids. Zhang et al. [11] present the current state of *Cyperus esculentus* L. including the allelopathic potential and antibacterial, antioxidant and insecticidal activities of this plant. Kato-Noguchi and Kato [12] give an overview of *Solidago canadensis* L. and *Solidago altissima* L. allelopathy, two species which are aggressive invaders in many parts of the world. Tabaglio et al. [13] thematize properties and microorganisms possibly involved in the resilience of velvetleaf (*Abutilon theophrasti* Medik.) against allelochemicals, such as benzoxazinoids. Casanova et al. [14] reviewed the great variety of allelochemicals found in microalgae. These compounds may be valuable to biocontrol weeds, insects, phytopathogenic fungi and bacteria.

This special issue demonstrates that the sustainable use of allelopathy in agriculture demands in-depth knowledge about the bioactive molecules responsible for influencing the complex interactions between organisms. It is imperative that microorganisms must be considered in allelopathic interactions. This knowledge is the first step in designing smart agroecosystems that ensure both the sustainable intensification of agricultural production, as advocated by the FAO to meet global food demands and combat poverty, and the protection of the environment and species diversity, as outlined in the European Green Deal.
